# Fine mapping of *qBK1*, a major QTL for bakanae disease resistance in rice

**DOI:** 10.1186/s12284-019-0295-9

**Published:** 2019-05-14

**Authors:** Sais-Beul Lee, Namgyu Kim, Yeon-Jae Hur, Su-Min Cho, Tae-Heon Kim, Ji-youn Lee, Jun-Hyeon Cho, Jong-Hee Lee, You-Chun Song, Young-Su Seo, Jong-Min Ko, Dong-Soo Park

**Affiliations:** 1 National Institute of Crop Science, Milyang, 50424 Republic of Korea; 2 Department of Microbiology, Pusan National University, Pusan, 46241 Republic of Korea

**Keywords:** Rice, Bakanae, *Gibberella fujikuroi*, QTL mapping, Resistance, Candidate gene

## Abstract

**Background:**

Bakanae disease is an important fungal disease caused by *Gibberella fujikuroi*. Incidence of rice bakanae disease creates serious problems in the foremost rice growing countries, and no rice variety has been found to be completely resistant to this disease. However, breeding rice varieties resistant to bakanae disease may be a cost-saving solution preferable to the application of fungicides. In this study, we aimed to determine the exact position and the candidate gene for *qBK1*, a major resistant quantitative trait locus (QTLs) for bakanae disease.

**Results:**

The genotypes/phenotypes of recombinants selected from backcrossed recombinant inbred lines of two rice varieties, Shingwang (resistant) and Ilpum (susceptible), indicated that the locus *qBK1*, conferring resistance to bakanae disease in Shingwang, was delimited to a 35-kb interval delimited by InDel 18 (23.637 Mbp) and InDel 19–14 (23.672 Mbp). Sequence analysis of this 35-kb region revealed four candidate genes, LOC_Os01g41770, LOC_Os01g41780, LOC_Os01g41790, and LOC_Os01g41800. There were many non-synonymous SNPs in LOC_Os01g41770 and the transcript of LOC_Os01g41790 was early terminated in Shingwang, whereas there were no differences in both LOC_Os01g41780 and LOC_Os01g41800 sequences between Ilpum and Shingwang. Expression profiling of the four candidate genes showed the up-regulation of LOC_Os01g41770, LOC_Os01g41780, and LOC_Os01g41790 in Ilpum and of LOC_Os01g41800 in Shingwang after inoculation of *G. fujikuroi.*

**Conclusion:**

Utilization of marker-assisted selection (MAS) with a precise molecular marker on *qBK1* could provide an effective tool for breeding rice varieties resistant to bakanae disease. To our knowledge, this is the first report on fine mapping and candidate gene approaches for identifying the gene for *qBK1*.

**Electronic supplementary material:**

The online version of this article (10.1186/s12284-019-0295-9) contains supplementary material, which is available to authorized users.

## Background

Bakanae disease (from the Japanese “foolish seedling”), caused by the fungus *Gibberella fujikuroi*, was first described in 1828 in Japan (Ito and Kimura 1931). It is one of the most serious and widespread problems of rice growing countries in Asia and Africa, and in North America and Italy (Ou 1985; Pra et al. 2010). The typical symptoms are abnormal elongation, including tall, lanky tillers, pale green flag leaves, dried-up leaves, and infertile panicles (Ou 1985; Mew and Gonzales 2002). Seeds contaminated with this fungus provide initial foci for secondary infection. Under favorable environmental conditions, infected plants have the capacity to produce numerous conidia that subsequently infect proximate healthy plants, resulting in major yield loss (Ou 1985; Rosales and Mew 1997). Bakanae disease decreases rice grain yield up to 50% in Japan (Ou 1985) and to 95% in India (Sunder et al. 1997; Fiyaz et al. 2014; Gupta et al. 2015). Germinating rice seeds in seed boxes for mechanical transplantation has caused many problems associated with diseases (Rosales and Mew 1997) including bakanae disease, which are not considered serious in direct seeding. However, bakanae disease has become a serious problem in hybrid rice breeding, which involves the increased use of growing plants in seed beds (Li and Luo 1997; Yang et al. 2003). The most common management practice for bakanae disease is seed treatment using hot water or fungicides (Gupta et al. 2015; Lee et al. 2018). However, the hot water immersion method (Hayasaka et al. 2001) for seed disinfection proved ineffective on severely infected rice seeds, because the hot water does not reach the pericarp of rice seeds. The application of fungicides is also markedly ineffective for destroying the spores of this fungal pathogen, as it has been reported to be resistant to fungicides (Ogawa 1988; Park et al. 2009; Kim et al. 2010; Lee et al. 2011). Therefore, the cultivation of resistant varieties would be a more economic and effective way to control this disease.

Quantitative trait loci (QTLs) analysis with a precise bioassay system facilitates the discovery of resistance loci and provides valuable information for germplasm discovery and breeding strategies for developing resistant cultivars. Several QTLs for bakanae disease resistance have been mapped on the rice genome. Yang et al. (2006) identified two QTLs on chromosome 1 and chromosome 10 using the Chunjiang06/TN1 doubled haploid population. In our previous study (Hur et al. 2015), we identified a major QTL, *qBK1*, from the Korean *indica* variety Shingwang. *qBK1* is located within a 520-kb region of chromosome 1 between the simple sequence repeat (SSR) markers RM8144 (23.20 Mb) and RM11295 (23.72 Mb) with RM9 (23.32 Mb) as the peak marker. Lee et al. (2018) found another QTL, *qBK1*^*WD*^, which is located on the physical map between markers chr01_13542347 (13.54 Mb) and chr01_15132528 (15.13 Mb). Fiyaz et al. (2016) identified three QTLs (*qBK1.1, qBK1.2*, and *qBK1.3*) on chromosome 1 and one QTL (*qBK3.1*) on chromosome 3 from the Indian *indica* variety Pusa 1342. Fiyaz et al. (2016) discussed that *qBK1.1* and *qBK1* are likely the same QTL, as *qBK1.1* is located between markers RM9 (23.32 Mb) and RM11282 (23.34 Mb), and the both QTLs share RM9 marker.

Additional molecular markers and recombination events of *qBK1* could potentially be used for higher resolution mapping. Considering its great importance in rice breeding and the biological value of bakanae disease, the present study aimed to determine the exact position and the candidate gene for *qBK1*. The results obtained in this study might be used for identifying the gene responsible for resistance against bakanae disease.

## Results

### Fine mapping of *qBK1*

**Fig. 1:**
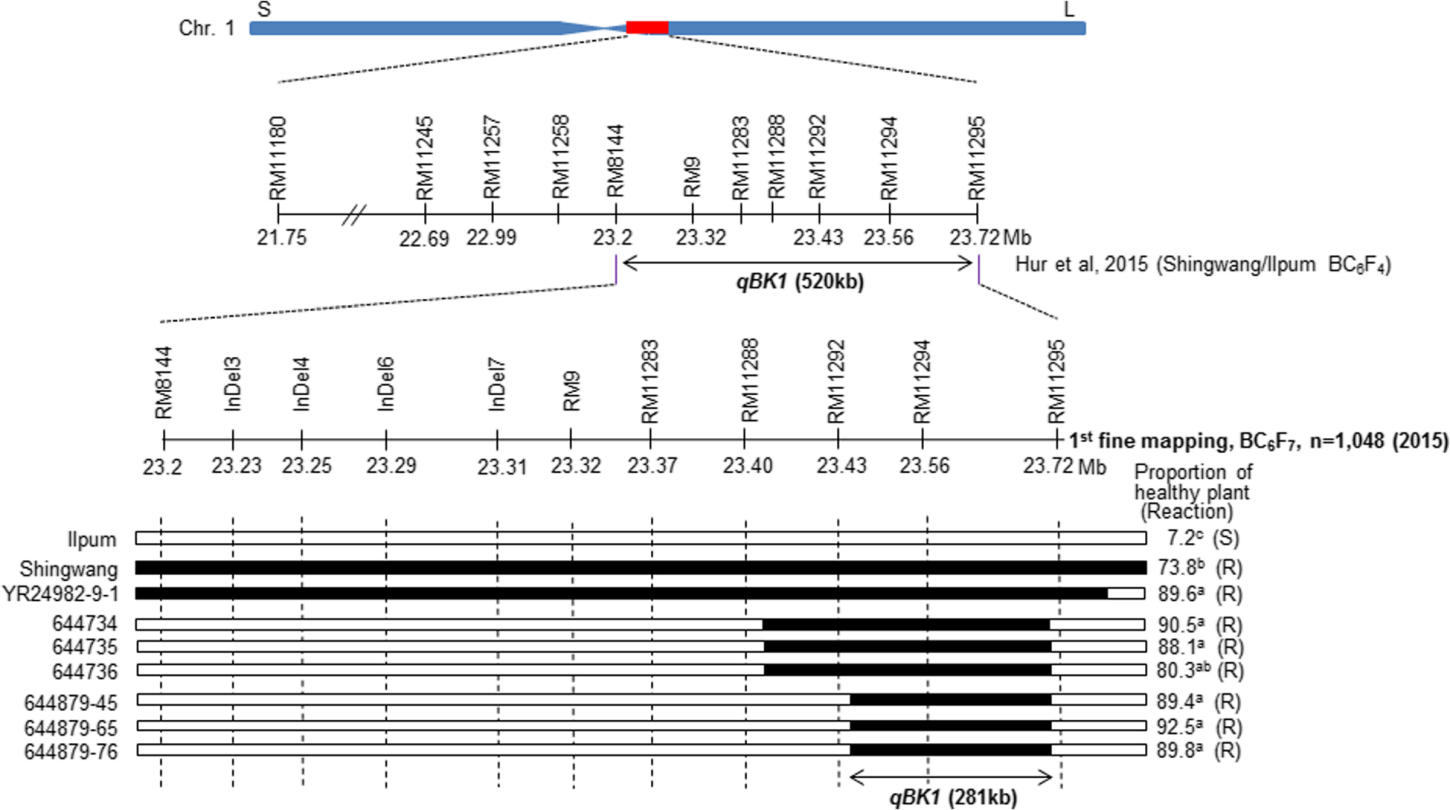
First fine mapping of *qBK1*. Genotype analysis and progeny test of homozygous recombinants in the region delimited by markers RM11292 and RM11295. YR28491–9-1 is the parental resistant BC_6_F_4_ near-isogenic line harboring *qBK1*. Black bars show the homozygous regions for Shingwang alleles; white bars indicate the homozygous regions for Ilpum alleles. R, resistant; S, susceptible

We previously identified *qBK1* in the 520 kb region between RM8144 and RM 11295 on chromosome 1 (Hur et al. 2015). Here we attempted to narrow down the position of *qBK1* using recombination events and additional molecular markers. A heterozygous BC_6_F_4_ line in the RM11292 to RM11295 region was selfed, and six lines harboring two different homozygous regions on the target position among the 1048 BC_6_F_7_ individual plants, were selected (Fig. 1). Three lines (644879–45, 644,879–65, and 644,879–76) harboring the resistance allele on RM11294 were resistant to bakanae disease. Based on this result, *qBK1* was delimited to a 281-kb interval between RM11292 and RM11295 in the first fine mapping (Fig. 1, Additional file 1: Figure S1).

**Fig. 2:**
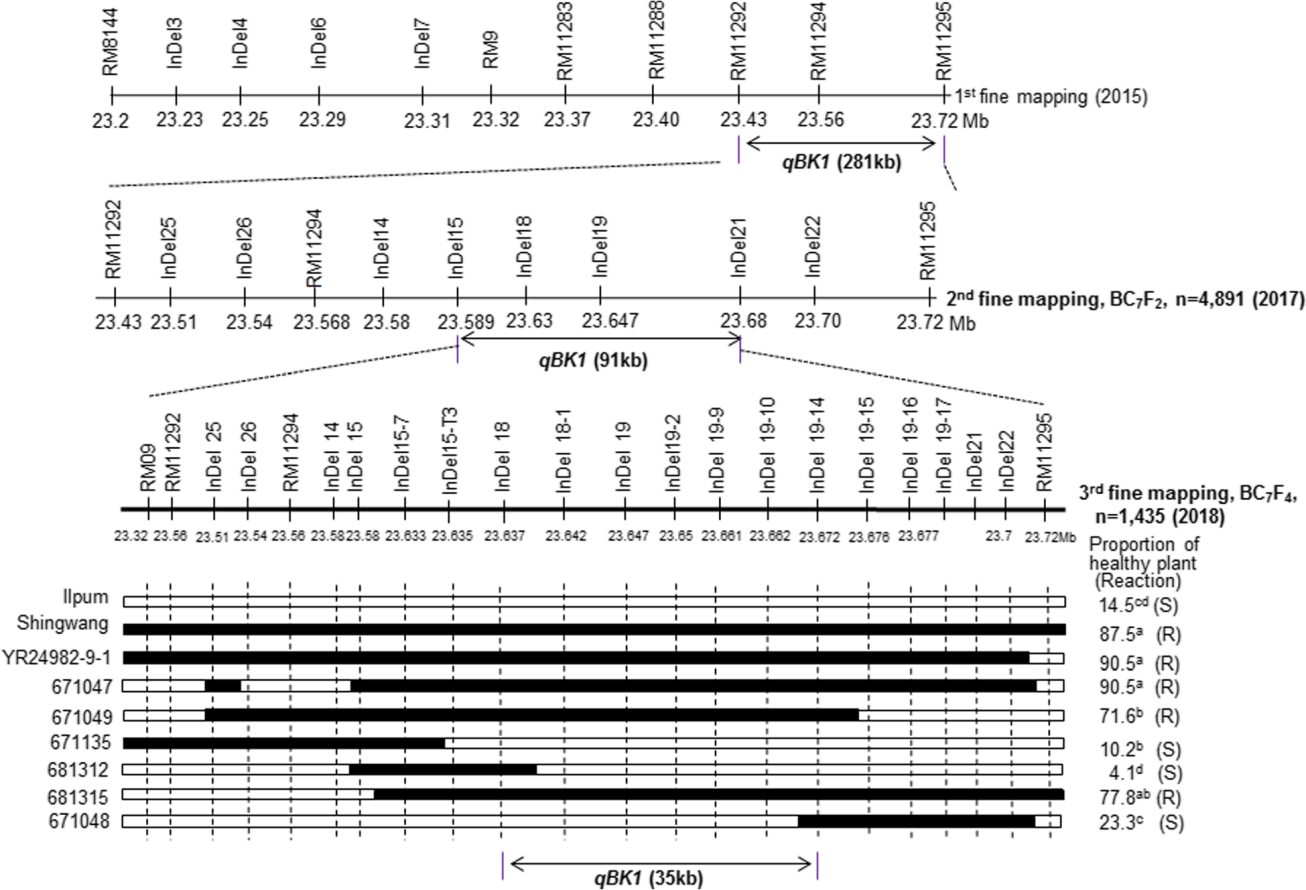
Second and third fine mapping of *qBK1*. Genotype analysis and progeny test of homozygous recombinants in the region delimited by the markers InDel 18 and InDel 19–14. YR28491–9-1 is the parental resistant BC_6_F_4_ near-isogenic line harboring *qBK1*. Black bars show the homozygous regions for Shingwang alleles; white bars indicate the homozygous regions for Ilpum alleles. R, resistant. S, susceptible

**Fig. 3:**
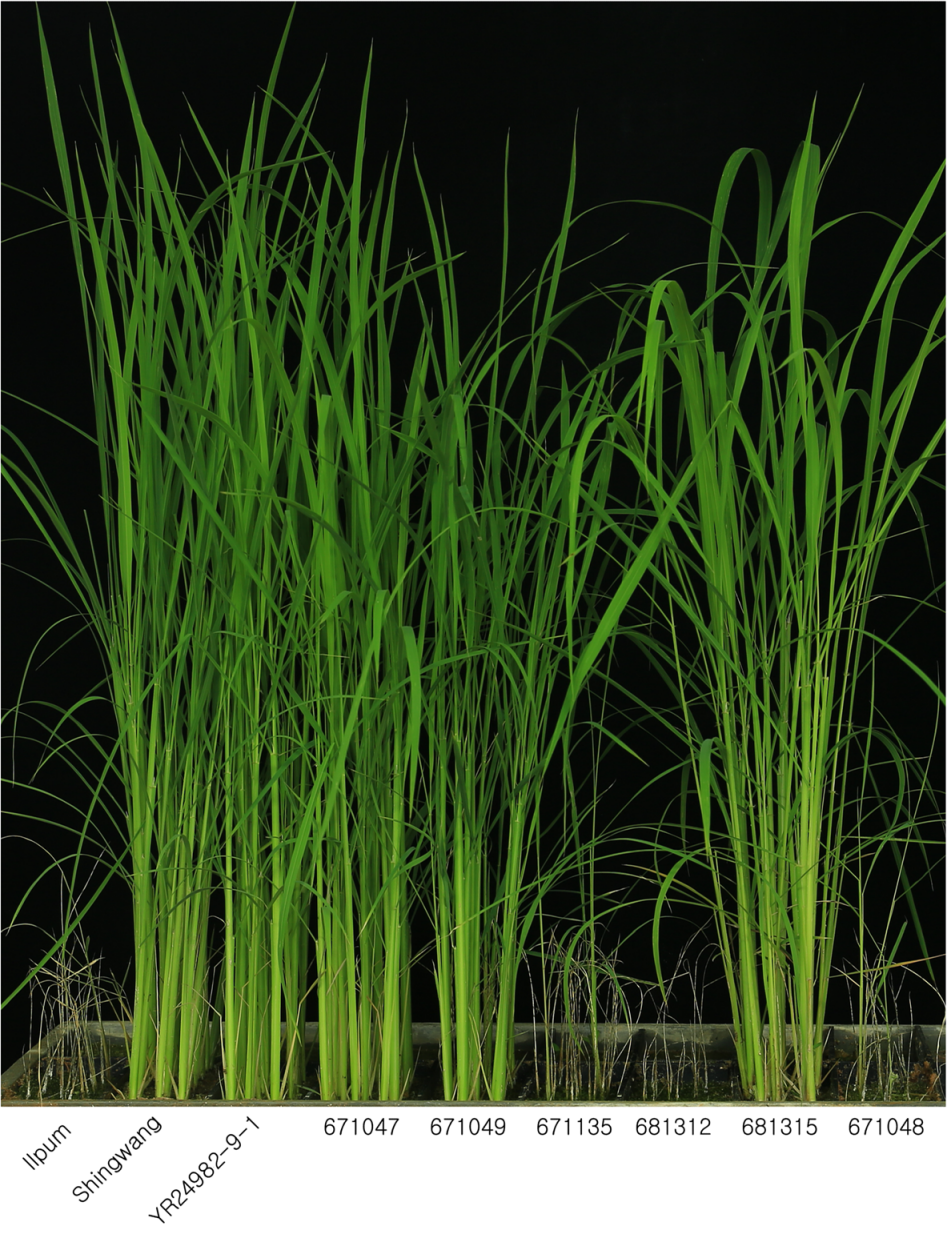
Phenotypic responses to bakanae disease in six homozygous recombinants in the third fine mapping of *qBK1*

A resistant line (644879–65) was further backcrossed with Ilpum (the susceptible parent), and genotypes of 4891 BC_7_F_2_ plants were analyzed using insertion-deletion (InDel) markers in the target region (RM11292 to RM11295) for the second fine mapping (Fig. 2, Fig. 3). In the second fine mapping, the target region of *qBK1* was delimited to 91-kb between InDel 15 and InDel 21 (data not shown). Seven homozygous recombinants, with three biological replicates each, were evaluated in the third fine mapping, which included two lines (671,047 and 671,049) used in the second fine mapping and other lines selected after analyzing 1485 BC_7_F_4_ lines harboring different recombination events in the target region.

The average proportions of Ilpum and Shingwang healthy plants were 14.5% and 87.5%, respectively. According to Duncan’s new multiple range test, Shingwang, YR28491–9-1, and lines 671,047, 671,049, and 681,315 were classified in Groups a and b, and regarded as resistant, while Ilpum and lines 671,135, 681,312, and 671,048, classified in Groups c and d, were regarded as susceptible. Based on the genotype and disease reaction of these recombinants, *qBK1* was delimited to a 35-kb interval by InDel 18 and InDel 19–14 (Fig. 2).

### Candidate gene analysis for *qBK1*

**Fig. 4:**
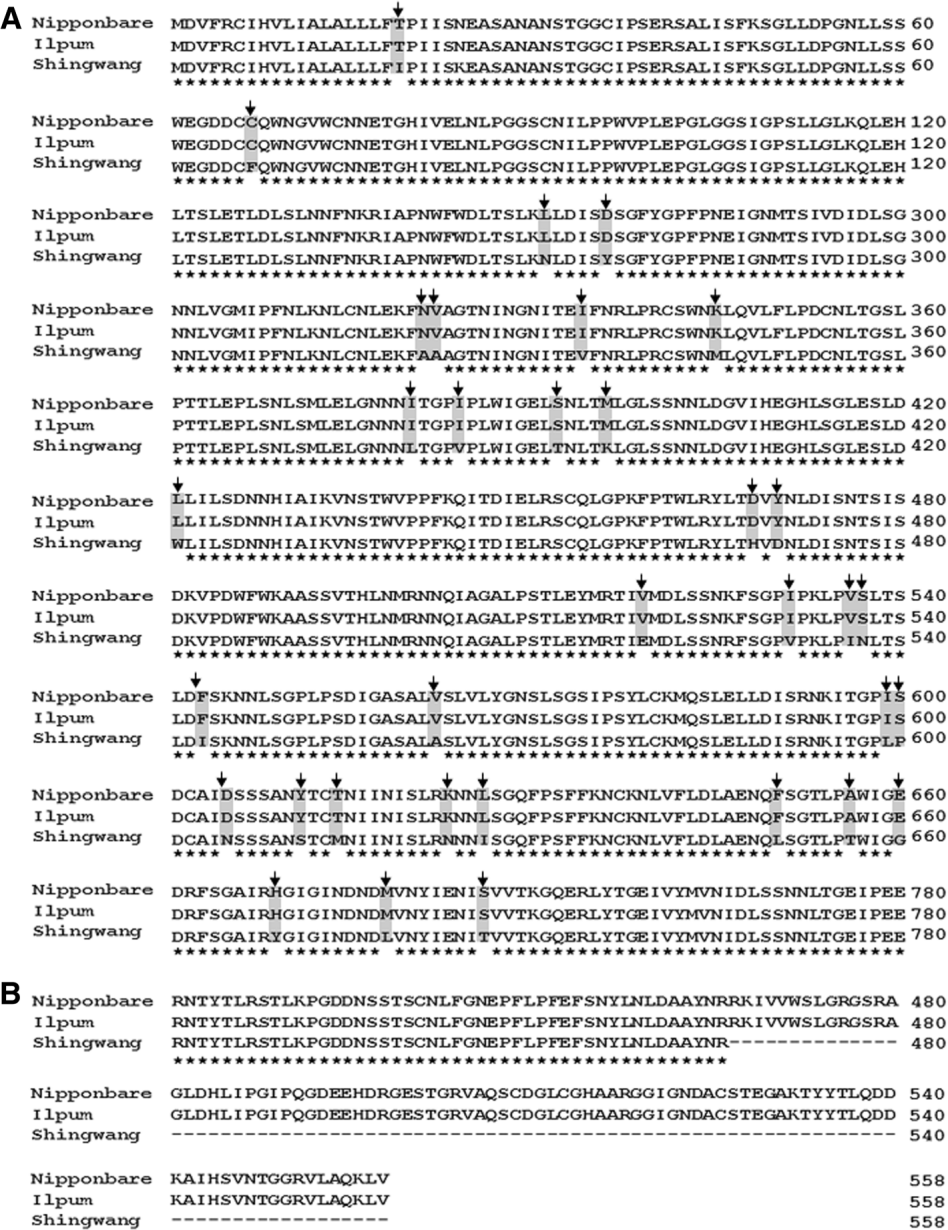
Multiple sequence alignment of LOC_Os01g41770 and LOC_Os01g41790 from Nipponbare, Ilpum and Shingwang. Putative amino acid sequences were translated from the entire cDNA sequences of LOC_Os01g41770 (**a**) and LOC_Os01g41790 (**b**) in Nipponbare, Ilpum and Shingwang rice varieties. Multiple sequence alignments were generated with CLUSTALW. Arrows represent single amino acid substitutions

Sequence analysis of the 35-kb region between InDel 18 and InDel 19–14, harboring the *qBK1* locus using the Rice Genome Annotation Project (http://rice.plantbiology.msu.edu/index.shtml) indicated that this region contained four candidate genes (Table 1). None of the four candidate genes had been reported as involved in bakanae disease resistance in rice. We isolated the cDNA of each of the four genes from Ilpum and Shingwang varieties by RT-PCR. Sequence analysis of the isolated genes revealed many base substitutions in LOC_Os01g41770 between susceptible (Ilpum) and resistant (Shingwang) varieties (Fig. 4a) and the transcript of LOC_Os01g41790 was early terminated in Shingwang compared to Ilpum (Fig. 4b), whereas there were no differences between LOC_Os01g41780 and LOC_Os01g41800 transcripts. Sequence analysis of LOC_Os01g41770 between the resistant susceptible varieties reveled 34 non synonymous SNPs among 65 base variations. Furthermore, the LOC_Os01g41790 sequence in the resistant variety had an early stop codon due to the insertion of 276 nucleotides in the terminal region.

**Table 1:** Candidate genes in the genomic region between InDel 18 and InDel 19–14 harboring the *qBK1* locus

Gene ID	Physical location (bp)	Putative function
LOC_Os01g41770	23,639,654– 23,642,880	Leucine rich repeat protein, putative, expressed
LOC_Os01g41780	23,648,265– 23,651,499	Leucine rich repeat protein, putative, expressed
LOC_Os01g41790	23,656,010–23,658,316	Expressed protein
LOC_Os01g41800	23,663,059– 23,664,444	Cytochrome P450 72A1, putative, expressed

**Fig. 5:**
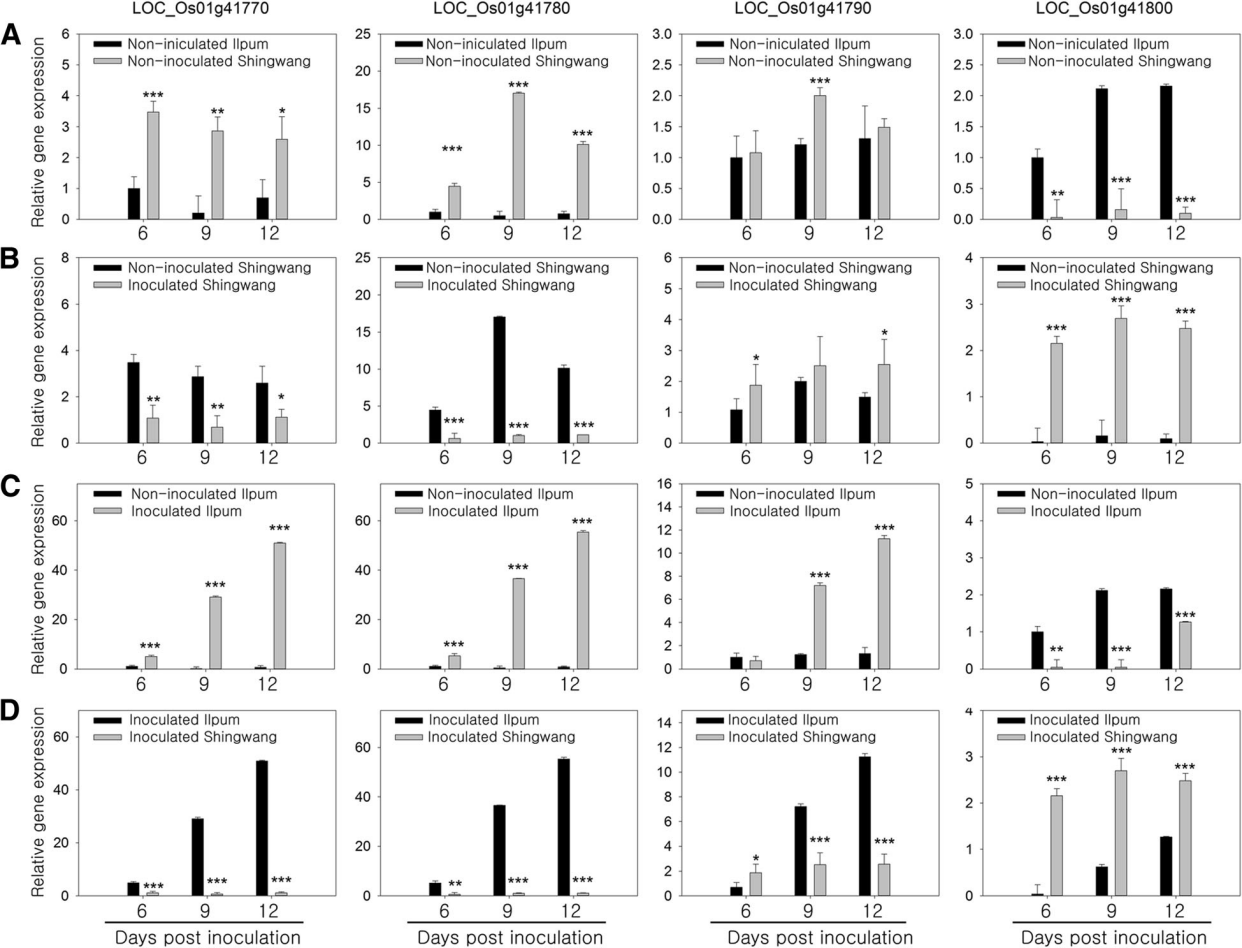
Relative expression of candidate genes in resistant and susceptible rice varieties inoculated with *Fusarium fujikuroi* isolate CF283 or non-inoculated. (A) Relative expression of candidate genes in non-inoculated Ilpum vs. Shingwang rice varieties. (B) Relative expression of candidate genes in non-inoculated vs. inoculated Shingwang. (C) Relative expression of candidate genes in non-inoculated vs. inoculated Ilpum. (D) Relative expression of candidate genes in inoculated Ilpum vs. Shingwang rice varieties. *Ubiqutin 5* (UBQ5) was used as the reference gene, and the data were calibrated relative to the expression of each gene in susceptible non-inoculated rice at 6 days post inoculation. Mean values followed by same letters are not significantly different according to Tukey’s HSD test (*P < 0.05, **P < 0.01, ***P < 0.001)

We analyzed the expression patterns of the four candidate genes in the bakanae disease resistant variety, Shingwang, and in the susceptible variety, Ilpum (Fig. 5). The relative expressions of LOC_Os01g41770 and LOC_Os01g41780 presented opposite patterns between the two rice varieties. The expression of LOC_Os01g41770 was higher in non-inoculated Shingwang than in non-inoculated Ilpum (Fig. 5a), and higher in non-inoculated Shingwang than in inoculated Shingwang (Fig. 5b). In contrast, the relative expression of LOC_Os01g41770 was much higher in inoculated than in non-inoculated Ilpum (Fig. 5c). The expression pattern of LOC_Os01g41780 was similar to that of LOC_Os01g41770, except for its high expression in non-inoculated Shingwang at 9 days post inoculation (dpi), and it maintained a higher expression than LOC_Os01g41770 (Fig. 5a, b, c). The relative expression of LOC_Os01g41790 between the non-inoculated Shingwang and Ilpum was similar, except at 9 dpi (Fig. 5a). However, after *G. fujikuroi* inoculation, both Shingwang and Ilpum showed a relative increase in the expression of LOC_Os01g41790 compared to non-inoculated groups at 6, 9, and 12 dpi (Fig. 5b, c). The expression pattern of LOC_Os01g41800 was opposite to that of LOC_Os01g41770 and LOC_Os01g41780. The expression of LOC_Os01g41800 was higher in inoculated Ilpum than in non-inoculated Shingwang (Fig. 5a), and higher in inoculated than in non-inoculated Shingwang (Fig. 5b). In addition, the relative expression of LOC_Os01g41800 was much higher in non-inoculated than in inoculated Ilpum (Fig. 5c).

The relative expressions of LOC_Os01g41770, LOC_Os01g41780, and LOC_Os01g41790 in inoculated Ilpum were higher than in inoculated Shingwang at 6, 9, and 12 dpi, whereas the expression of LOC_Os01g41800 was higher in inoculated Shingwang than in inoculated Ilpum (Fig. 5d).

## Discussion

### Fine mapping of *qBK1*, a major QTL of bakanae disease resistance

**Fig. 6:**
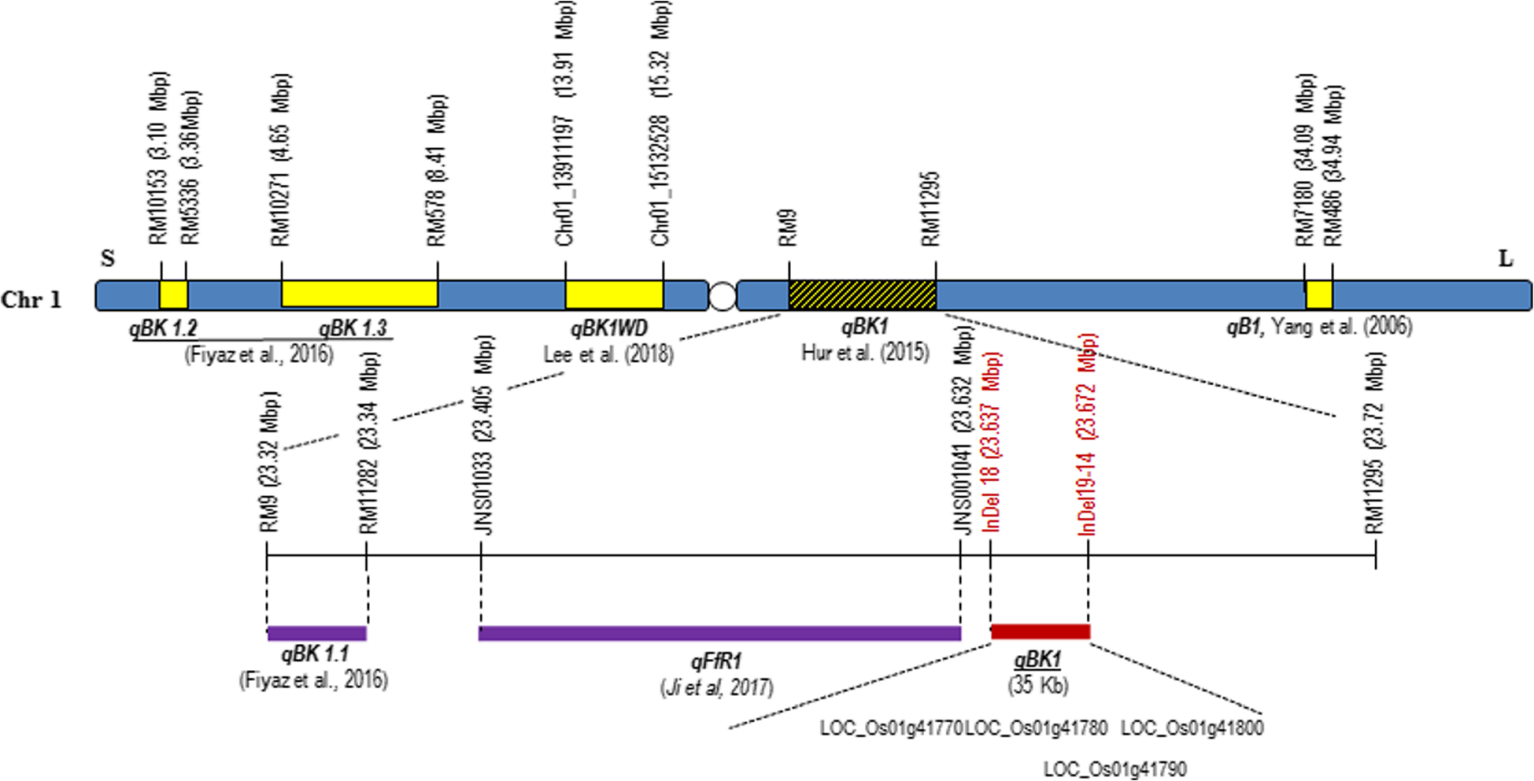
Physical location of bakanae disease resistance quantitative trait loci on chromosome 1

In this study, we performed the fine mapping of the *qBK1* locus related to bakanae disease resistance based on genotype and phenotype analyses of homozygous recombinants on the backcross progeny of Ilpum and Shingwang, using newly developed InDel and SNP markers. In addition to *qBK1*, six QTLs have been identified on chromosome 1 including *qBK1.2*/*qBK1.3*/*qBK1.1* from Pusa 1342 (Fiyaz et al. 2016), *qB1* from Chunjiang 06 (Yang et al. 2006), *qFfR1* from Nampyeong (Ji et al. 2017), and *qBK1*^*WD*^ from Wonseadaesoo (Lee et al. 2018). *qBK1_628091* (0.6 Mbp to 1.0 Mbp on chromosome 1) and qBK4_31750955 (31.1 Mbp to 31.7 Mbp on chromosome 4) were revealed by GWAS approach using *japonica* rice germplasm collection (Volante et al., 2017). Three QTLs (*qBK1*, *qBK1.1*, and *qFfR1*) were found in a similar region in spite of the different source of resistant varieties (Fig. 6). Fiyaz et al. (2016) mapped *qBK1.1* to a 200-kb region between markers RM9 and RM11282 from the Pusa 1121/Pusa1342 cross. These authors hypothesized that *qBK1.1* and *qBK1* (Hur et al. 2015) might be the same QTL as they had overlapping positions. Ji et al. (2017) found that QTL *qFfR1* was located in a 230-kb region of rice chromosome 1 in the Korean *japonica* variety Nampyeong, and suggested that the three QTLs *qBK1, qBK1.1*, and *qFfR1* might indicate the same gene. In the present study, the *qBK1* locus was narrowed to a 35-kb region between InDel 18 and InDel 19–14 in the 520-kb region on chromosome 1 identified in our previous study (Hur et al. 2015).

### Application of marker-assisted selection (MAS) for rice breeding

**Fig. 7:**
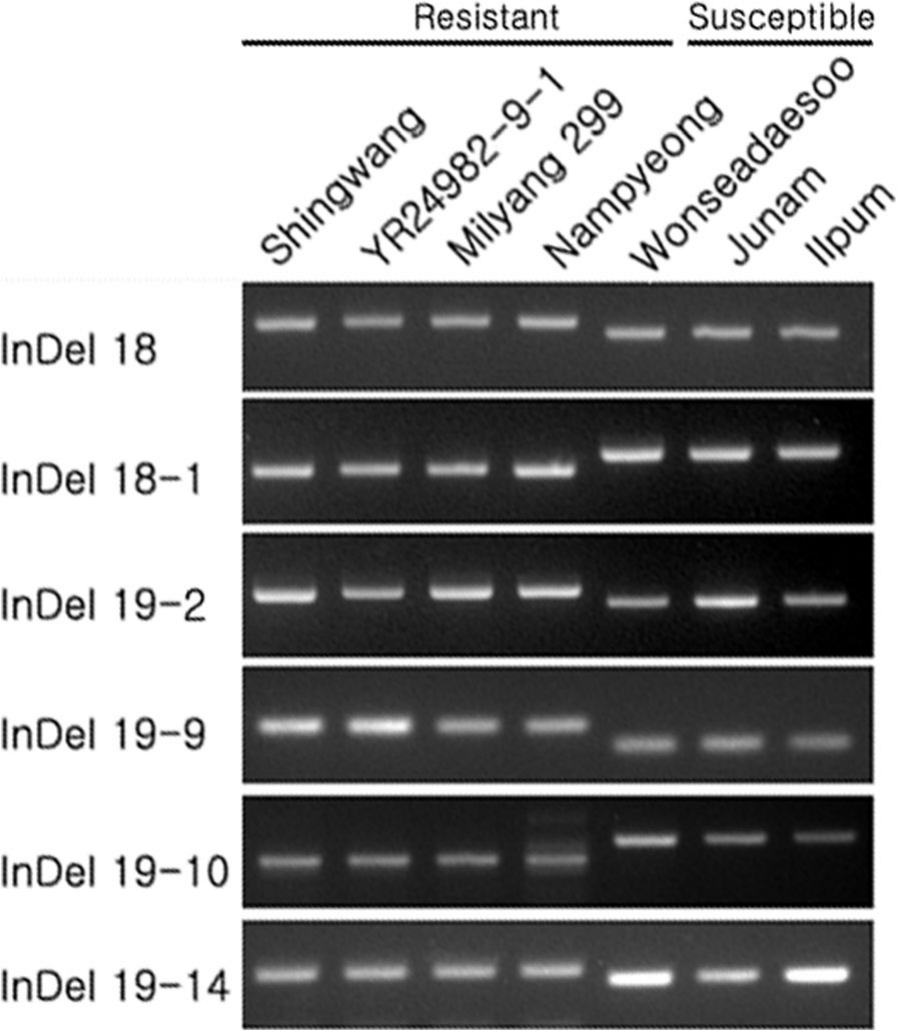
Genotype of resistant and susceptible rice varieties using the new InDel markers

We further analyzed whether each six InDel markers (InDel 18, InDel 18–1, InDel 19–2, InDel 19–9, InDel 19–10, and InDel 19–14) is proper for MAS to detect *qBK1* by adapting it to rice varieties with diverse sources of resistance (Fig. 7, Additional file 1: Figure S1). Varieties YR28491–9-1 and Milyang 299 harbor the *qBK1* originated from Shingwang. Resistant allele of these three resistant varieties were shared by that of Nampyeong, which harbors *qFfR1* (Ji et al. 2017), but different from those of Wonseadaesoo containing *qBK1*^*WD*^ (Lee et al. 2018). Thus, it is likely that *qBK1* and *qFfR1* are the same QTL. Lee et al. (2018) revealed that rice lines harboring *qBK1*^*WD*^ showed a higher level of resistance than those with *qBK1*. Furthermore, the pyramided rice lines harboring *qBK1*^*WD*^ + *qBK1* had a much higher level of resistance than those harboring either *qBK1*^*WD*^ or *qBK1*. Fiyaz et al. (2016) reported that *qBK1.2*, is a strong candidate for MAS of bakanae resistance, as it showed higher LOD (logarithm of the odds) score and PVE (percentage of variance explained) than *qBK1.1* and *qBK3.1.*

Four *Fusarium* species from the *G. fujikuroi* species complex, including *F. andiyazi*, *F. fujikuroi*, *F. proliferatum*, and *F. verticillioides*, have been reported to be associated with bakanae disease (Wulff et al. 2010). The use of diverse genetic resistance sources and accurate molecular markers will be important for rice breeding programs aiming to develop varieties that have enhanced resistance to bakanae disease and to overcome the breakdown of resistance genes. Six InDel markers on *qBK1* presented in this study can be used in PCR-assisted selection of bakanae disease resistance in rice-breeding programs.

### Candidate gene analysis for *qBK1*

Fine mapping revealed four candidate genes in a 35-kb region of *qBK1* (Table 1, Fig. 6). Both LOC_Os01g41770 and LOC_Os01g41780 encode putative leucine rich repeat (LRR) receptor-like proteins. The sequences of transcript LOC_Os01g41770 in the bakanae disease resistant variety (Shingwang) differed from that in the susceptible variety (Ilpum) in 34 amino acids, whereas those of LOC_Os01g41780 were identical. These two genes showed very similar expression patterns in Shingwang and Ilpum before and after inoculation of *F. fujikuroi*, and showed relatively higher expressions in Shingwang than in Ilpum under the non-inoculation condition (after mock inoculation). However, both genes showed down-regulated expression in Shingwang, but extremely up-regulated expression in Ilpum after inoculation. Based on the expression profiling of LRR receptor like-proteins, these two candidate genes might be involved in the negative regulation of disease resistance. However, these two genes might also act as positive regulators. For example, tomato Ve1, a LRR receptor like-protein contributing to *Verticilium* wilt resistance, showed no difference in expression patterns between resistant and susceptible tomato varieties (Fradin et al. 2009). Although most of the known LRR receptor like-proteins play diverse roles in resistance to pathogens, LRR receptor kinase BIR3 was recently identified as a negative regulator involved in brassinosteroid-dependent development and innate immunity through the inhibition of BAK1 complex formation and stabilization in Arabidopsis (Imkampe et al. 2017). Thus, LOC_Os01g41770 and/or LOC_Os01g41780 might be involved in either positive or negative regulation of bakanae disease resistance. The transcript of LOC_Os01g41790, which is unknown about functional annotation in both genotypes, was early terminated in resistant variety. Although LOC_Os01g41790 was induced both susceptible and resistant varieties after *G. fujikuroi* inoculation, the differential induction levels may give specific function in susceptible and resistant varieties (Fig. 5d). In contrast, LOC_Os01g41800, encoding a putative cytochrome P450 monooxygenase, exhibited up-regulated expression in the resistant variety, Shingwang and down-regulated expression in the susceptible variety, Ilpum. Plant cytochrome P450 monooxygenases are a group of redox proteins that catalyze various oxidative reactions mediating plant defense, including the synthesis and metabolism of many chemical compounds that are related to plant defense against a range of pathogenic microbes and insects (Isin and Guengerich 2007). Like LOC_Os01g41800, the grape cytochrome P450 monooxygenase CYP736B transcript was up-regulated in Pierce’s disease (PD)-resistant plants and down-regulated in PD-susceptible plants six weeks after *Xylella fastidiosa* inoculation (Cheng et al., 2010) Thus, LOC_Os01g41800 appears to be a positive regulator of bakanae disease resistance based on the analysis of its expression pattern.

## Conclusions

Introducing *qBK1* along with the utilization of MAS could provide tools for breeding rice varieties resistant to bakanae disease. The results from fine mapping of *qBK1* and the information on the four candidate genes obtained here will be useful to determine the gene on the *qBK1* QTL by using additional molecular markers and transformation studies.

## Methods

### Plant materials

Shingwang, the bakanae disease resistant *indica* variety, was selected from large-scale screening of rice germplasms (Kim et al. 2014). The QTL *qBK1* was identified from BC_6_F_4_ near-isogenic lines resulting from Shingwang (donor) and Ilpum (recurrent parent) crosses (Hur et al. 2015). In the present study, we generated further segregating populations using selected heterozygous plants in a targeted region to narrow down the position of *qBK1* (Fig. 1).

### Development of InDel and SNP markers

The number of polymorphic SSR markers was not sufficient to narrow down the location of *qBK1* in the segregating populations. Twelve InDel markers (Additional file 2: Table S1) were therefore designed by analyzing the sequence differences between Nipponbare (Gramene database; http://www.gramene.org) and 93–11 (BGI-RIS; http://rice.genomics.org.cn) in the region between RM8144 (23.20 Mb) and RM9 (23.32 Mb). Each InDel marker was designed to produce PCR products of 250–350 bp with fragment size differences of 15–25 bp, which are suitable for detection in agarose gels. Genomic DNA sequences of the targeted region were amplified from Ilpum and Shingwang individuals by PCR and sequenced in an ABI PRISM 3730XL analyzer (Applied Biosystems, Foster City, CA, USA). For fine mapping, eight additional InDel markers (Additional file 2: Table S2) and a SNP marker (Additional file 2: Table S3) for ARMS(Amplification-refractory mutation system) PCR was designed based on the sequence differences between Ilpum and Shingwang in the based on the initial 520-kb region between SSR markers RM8144 (23.20 Mb) and RM11295 (23.72 Mb), as described in Hur et al. (2015). The PCR cycling conditions for the InDel and SSR markers were 2 min at 94 °C, followed by 35 cycles at 94 °C for 20 s, 55 to 60 °C for 40 s, and 72 °C for 30 s, and a final extension for 7 min at 72 °C. The PCR cycling condition for the SNP marker for ARMS PCR was 2 min at 94 °C, followed by 35 cycles at 94 °C for 20 s, 55 to 65 °C for 40 s, and 72 °C for 1 min, and a final extension for 7 min at 72 °C. The amplified products were separated using a 3% agarose gel electrophoresis and visualized with ethidium bromide.

### Evaluation of bakanae disease resistance

The evaluation of bakanae disease was performed using a method modified from that described by Kim et al. (2014) and Lee et al. (2018). The *F. fujikuroi* isolate CF283 was inoculated in potato dextrose broth and cultured at 26 °C under continuous light for 1 week. The fungal spore concentration was adjusted to 1 × 10^6^ spores/mL using a hemocytometer to obtain standardized inoculums. Forty seeds per line were placed into a tissue-embedding cassette (M512, Simport, Beloeil, QC, Canada), surface sterilized in a hot water bath (57 °C) for 13 min, and allowed to drain before they were soaked in a conidial suspension in another tray for 3 days at 26 °C with gentle shaking four times per day. After inoculation, 30 seeds per line were sown in nursery bed soil in a seedling tray. The inoculated seedlings were grown in a greenhouse at 28 ± 5 °C during the day and 23 ± 3 °C at night, in a 12 h light/dark cycle. The response to bakanae disease was evaluated by calculating the proportion of healthy plants in a given plot 1 month after sowing. Healthy and unhealthy plants were classified by the method described by Kim et al. (2014). Plants with the same phenotype as untreated plants or slight elongated seedlings with no thinness or yellowish coloring after infection were regarded as healthy plants.

### Quantitative real-time(qRT) PCR

Total RNA was extracted from shoots and leaves of Ilpum and Shingwang at 6, 9, and 12 days after bakanae disease infection with *F. fujikuroi* isolate CF283 using TRizol reagent (Thermo Fisher Scientific, Waltham, MA, USA). The cDNA libraries were then constructed from total RNA samples (2 μg per sample) using SuperScript III reverse transcriptase (Thermo Fisher Scientific) following the manufacturer’s instructions. Primer sets (Table 2) for generating specific PCR products on the conserved region of each candidate genes (LOC_Os01g41770, LOC_Os01g41780, LOC_Os01g41790, LOC_Os01g41800) were designed to anneal similar temperature using Primerplus 3 (https://primer3plus.com/), except for the *ubiqutin 5* (UBQ5; reference gene) primer set (Jain et al. 2006). The qRT-PCR was performed using the Rotor-Gene Q (Qiagen, Hilden, Germany) and TOPreal™ qPCR 2× PreMIX SYBR Green with low ROX (Enzynomics, Inc., Daejeon, Korea) using 100 ng of cDNA library as template. The conditions of the qRT-PCR reactions were: 10 min at 95 °C, followed by 45 cycles of 95 °C for 10 s, 60 °C for 15 s and 72 °C for 15 s. The conditions of the melting curve analysis were 72–95 °C, increased by 1 °C per second *s*^-1^. Relative transcript levels were calculated by the 2^-ΔΔ*C*T^ method (Livak and Schmittgen 2001) on the Rotor-Gene Q software (Qiagen).

**Table 2:** Candidate genes primer sets for quantitative RT-PCR

Primer ID	Forward primer (5′-3′)	Reverse primer (5′-3′)
LOC_Os01g41770	5′ AGAAACAAGATAACTGGGCCA 3’	5′ TAAGAATACCAAAGAAGGTA 3’
LOC_Os01g41780	5′ AATTCCCTAGCTTCCTTC 3’	5′ ACTTCCTGATATGTTGTTATG 3’
LOC_Os01g41790	5′ CGGTGAAGACCAGGATTTGT 3’	5′ ATGGATCCTCTCATGGCAAG 3’
LOC_Os01g41800	5′ TTGTTCATCCACCATGATCC 3’	5′ ACGCCTAAGCCTGCTGGTT 3’
UBQ5(ubiquitin 5)	5′ ACCACTTCGACCGCCACTACT 3’	5′ ACGCCTAAGCCTGCTGGTT 3’

## Additional files

Additional file 1: **Figure S1.** Phenotypic responses to bakanae disease in six homozygous recombinants for first fine mapping of *qBK1*. (TIF 11093 kb)

Additional file 2: **Table S1.** Twelve InDel markers designed by analyzing sequence differences between Nipponbare and 93–11. **Table S2.** Seven InDel markers designed by analyzing the differences between Ilpum and Shingwang sequences. **Table S3.** SNP marker for ARMS PCR designed by analyzing the differences between Ilpum and Shingwang sequences. (DOCX 18 kb)

## Abbreviations

InDel: insertion-deletion
LOD: logarithm of the odds
MAS: marker-assisted selection
PVE: percentage of variance explained
qRT-PCR: quantitative RT-PCR
QTLs: quantitative trait loci
SSR: simple sequence repeat
UBQ5: *ubiqutin 5*
