# Supplementary figures and images for: Fine mapping of qBK1, a major QTL for bakanae disease resistance in rice
Source: Rice (N Y). 2019 May 14;12:36. doi: 10.1186/s12284-019-0295-9 (PMC6517470; doi:10.1186/s12284-019-0295-9)

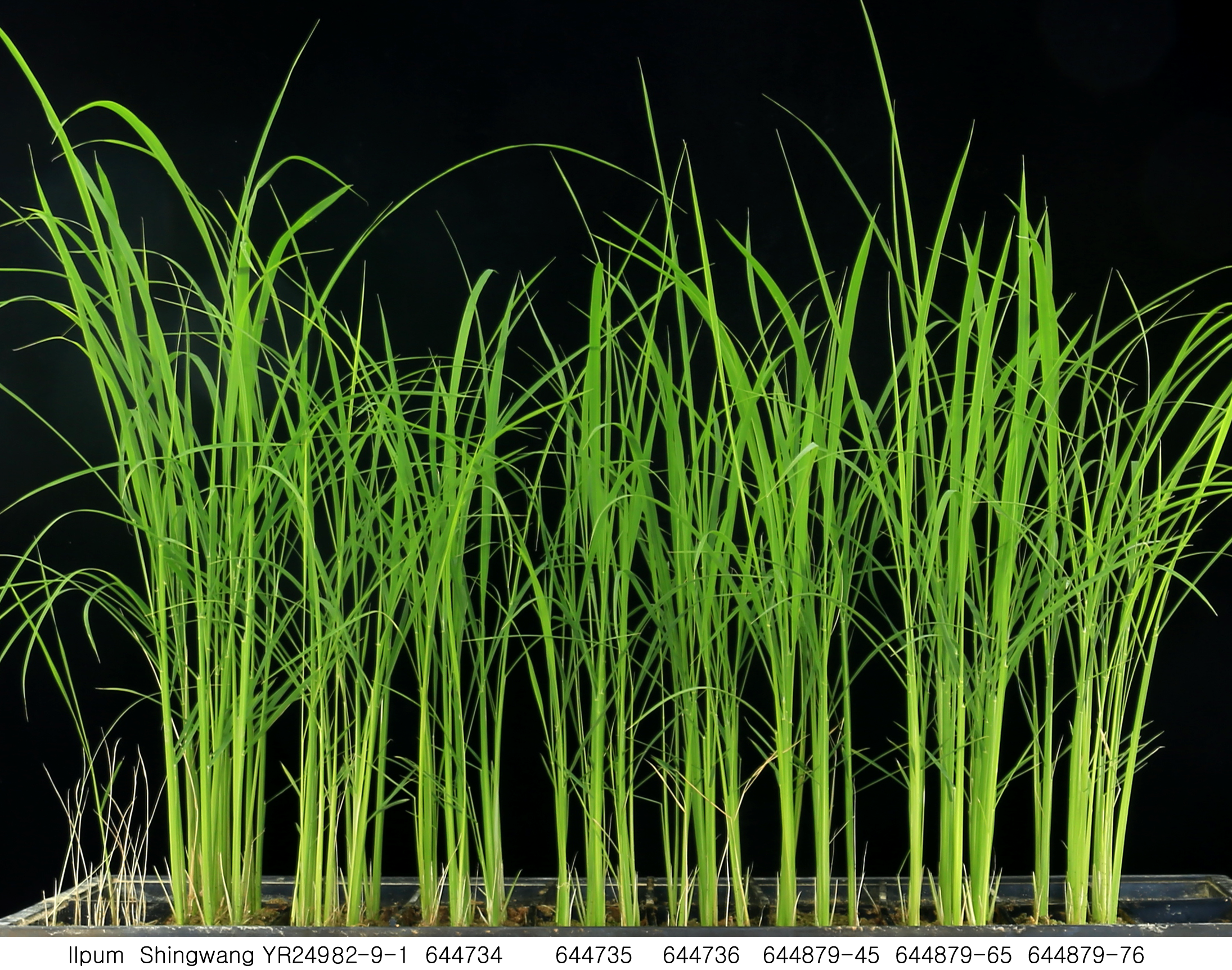

Supplement: Supplementary file 1 — Figure S1. Phenotypic responses to bakanae disease in six homozygous recombinants for first fine mapping of qBK1. (TIF 11093 kb) [file 12284_2019_295_MOESM1_ESM.tif]
