# Supplementary material for: Fine mapping of qBK1, a major QTL for bakanae disease resistance in rice
Source: Rice (N Y). 2019 May 14;12:36. doi: 10.1186/s12284-019-0295-9 (PMC6517470; doi:10.1186/s12284-019-0295-9)
Supplement: Supplementary file 2 — Table S1. Twelve InDel markers designed by analyzing sequence differences between Nipponbare and 93–11. Table S2. Seven InDel markers designed by analyzing the differences between Ilpum and Shingwang sequences. Table S3. SNP marker for ARMS PCR designed by analyzing the differences between Ilpum and Shingwang sequences. (DOCX 18 kb) [file 12284_2019_295_MOESM2_ESM.docx]

Table S1. Twelve InDel markers designed by analyzing sequence differences between Nipponbare and 93-11

| Primer ID | Forward primer (5'-3') | Reverse primer (5'-3') |
| --- | --- | --- |
| InDel 3 | GGTAATGGAGGCAAGTTTTGG | CCAAGGAGATTTGCAACCTG |
| InDel 4 | TGTGAAGGTGGAGAGTTCGG | TTCGTGCGAGTCATGGTGAT |
| InDel 5 | TCGCTACGTAAGACTGGTTG | CAAGCTTCCATTGGTGTTCG |
| InDel 7 | GCTTCGGGAATGGTATAGGC | CGATAAATGCTTCCACCACG |
| InDel 14 | GCTCGGGTGAAAATTGAGGT | TCTTGCAGGAAGGCGTATTG |
| InDel 15 | GTGCAGCTACTGCTCCCAAA | TGGGGATCGGGATTAGACTC |
| InDel 18 | GGCTAGGTCTCGGGTTTGAG | CTGTGGATGGGCCATAAAAC |
| InDel 19 | ACATTGCTAAATGCACACAAACA | CATGAAACAGTTTAACAGGGCA |
| InDel 21 | GGCTGAGAACGGTTGTATGG | TGGATGTGTCCTGACTATTTTGG |
| InDel 22 | ATGAAACTCGACCCCTGGAG | ATATGGTGGTGGGGTTGGTT |
| InDel 25 | GCACTGCACCCACATTGATT | GAATTGTGAGATGCGAGCGT |
| InDel 26 | CAGATGCGGAGAGAAACGTG | GAATGCCATAATCCGACCCT |

Table S2. Eight InDel markers designed by analyzing the differences between Ilpum and Shingwang sequences

| Primer ID | Forward primer (5'-3') | Reverse primer (5'-3') |
| --- | --- | --- |
| InDel15-7 | CAAGCGGTACTGGACCACTG | GAATTCTTTGGAAGAGTTGA |
| InDel18-1 | GGAGGAGCTTCTATCTGCTG | TAACACCACCGAACATCTTG |
| InDel19-2 | GAAAGATCAAGATAACTGAG | ACAACTGGTTTTGGGATCTC |
| InDel19-9 | GAGATGCACATGGAACTAAG | AGGATGGAGATATGTGACGA |
| InDel19-10 | TCTACGTCACGGTTGGATCC | CGGCGGCGACGACCGTCCCT |
| InDel19-14 | CAGAGTGCCCCAATGGAACA | GTTGCCGCACCCGACGCCCT |
| InDel19-16 | GTACAGCTAAACCCTGGATT | AAGGTTCGGTCTAAAGTTCT |
| InDel19-17 | ATGTATTCCTCTGTAACTCG | AGCAACATGATCATAGCGGT |

Table S3. SNP marker for ARMS PCR designed by analyzing the differences between Ilpum and Shingwang sequences

| Primer ID | Outer/Inner | Forward primer (5’-3’) | Reverse primer (5’-3’) |
| --- | --- | --- | --- |
| InDel 15-T3 | Outer | CTGTTGAACCTAATTTCACA | TCACACATCCCCTCATACTC |
|  | Inner | ATCTTTAGCGTGGGGGATCA | TGATAGGGAGCCTCCTGCCA |
